# Flexibility transition and guest-driven reconstruction in a ferroelastic metal–organic framework†

**DOI:** 10.1039/c4ce01572j

**Published:** 2014-10-02

**Authors:** Sarah J. Hunt, Matthew J. Cliffe, Joshua A. Hill, Andrew B. Cairns, Nicholas P. Funnell, Andrew L. Goodwin

**Affiliations:** Department of Chemistry, University of Oxford, Inorganic Chemistry Laboratory South Parks Road Oxford OX1 3QR UK andrew.goodwin@chem.ox.ac.uk

## Abstract

The metal–organic framework copper(i) tricyanomethanide, Cu(tcm), undergoes a ferroelastic transition on cooling below *T*_f_ = 240 K. Thermal expansion measurements reveal an order-of-magnitude variation in framework flexibility across *T*_f_. The low-temperature phase α-Cu(tcm) exhibits colossal positive and negative thermal expansion that is the strongest ever reported for a framework material. On exposure to acetonitrile, Cu(tcm) undergoes a reconstructive solid-phase transition to acetonitrilocopper(i) tricyanomethanide. This transition can be reversed by heating under vacuum. Infrared spectroscopy measurements are sensitive to the phase change, suggesting that Cu(tcm) may find application in solid-phase acetonitrile sensing.

## Abstract 2

Copper(i) tricyanomethanide shows exceptional mechanical and coordination flexibility in response to temperature and guest sorption.

## Graphical abstract

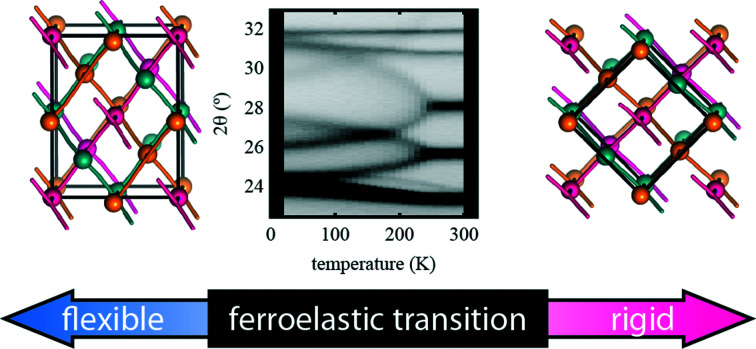

## 1. Introduction

**Fig. 1:**
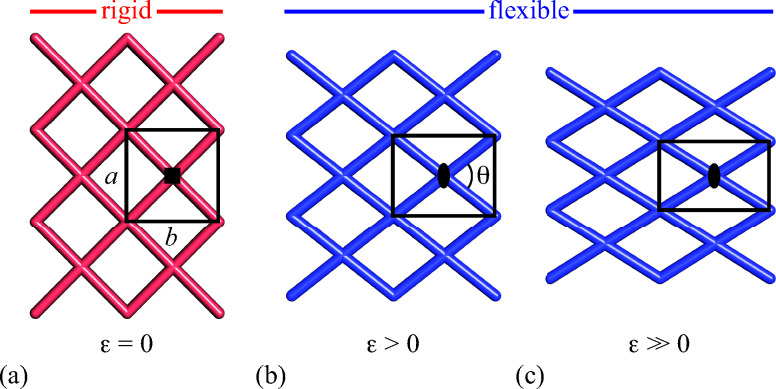
The dominant low-energy deformation mode of ‘wine-rack’ framework materials involves ‘breathing’ *via* changes in the framework angle *θ*. For the ideal square geometry in (a) this deformation is forbidden by the presence of fourfold rotation axes. Consequently the change in angle from square (a) to rectangular (b) geometries represents a symmetry-breaking ferroelastic phase transition, for which the deviation of the angle *θ* from 90° is related to the order parameter *ε* = (*b* − *a*)/(*a* + *b*). Breathing of the lower-symmetry rectangular geometry is allowed by the remaining symmetry elements (such as the diads shown here), such that the progression from geometries (b) to (c), for example, is continuous in all respects.

Framework materials with the wine-rack topology have emerged as an important class of materials because their characteristic low-energy ‘breathing’ mode allows for extreme mechanical responses to external stimuli.^1–3^ Examples include sorption- and temperature-driven breathing in the so-called MIL family of metal–organic frameworks (MOFs),^4,5^ and colossal positive/negative thermal expansion (PTE/NTE) and negative linear compressibility (NLC) in the transition-metal dicyanometallates.^6–8^ In all cases framework breathing couples expansion in one direction to contraction in a perpendicular direction, with the framework angle *θ* approaching 90° for increased framework volumes [Fig. 1]. While it is usually symmetry-allowed for these systems to adopt arbitrary geometries (*i.e.* values of *θ*), in some cases the *θ* = 90° limit actually corresponds to a higher space group symmetry.^9^ For this square geometry, framework breathing is symmetry forbidden, resulting in a fundamental increase in rigidity. So the existence of a ‘flexibility transition’ between low- and high-symmetry geometries would allow wine-rack frameworks to be switched between compliant (low-symmetry, *θ* ≠ 90°) and rigid (high-symmetry, *θ* = 90°) states.

Such transitions are well established in the field of conventional ceramics, where they are usually discussed in the context of ferroelasticity.^10–13^ The magnitude of symmetry-breaking strain *ε* [= (*b* − *a*)/(*a* + *b*) for a tetragonal–orthorhombic transition] acts as an order parameter just as magnetisation does for ferromagnets and polarisation for ferroelectrics. In this way, the rigid high-symmetry ‘paraelastic’ state of Fig. 1 corresponds to *ε* = 0 and the flexible low-symmetry ‘ferroelastic’ state to *ε* > 0. The crucial difference between MOF-type systems and conventional ceramics is that the extreme mechanical flexibilities of the former allow, in principle, for very much larger strains to develop across a ferroelastic transition. Consequently the flexibilities of paraelastic and ferroelastic states may be substantially more different for these materials than is the case for conventional inorganics. While there are a handful of very recent studies of ferroelastic transitions in MOFs,^14,15^ and indeed switching between ‘narrow pore’ and ‘large pore’ states of pillared MOFs might be interpretable in terms of an improper ferroelastic response,^9^ there remains a clear need to establish a model system in which ferroelasticity drives a fundamental change in framework flexibility.

Here we show that the previously-unreported framework material copper(i) tricyanomethanide, Cu(tcm), exhibits a ferroelastic transition at a critical temperature *T*_f_ = 240 K. Under ambient conditions, this system adopts a tetragonal wine-rack topology, for which breathing is symmetry forbidden. On cooling below 240 K the crystal symmetry lowers to an orthorhombic subgroup, for which breathing is symmetry allowed. Measurement of the coefficients of thermal expansion *α* allows quantification of the flexibility of both high- and low-temperature phases; we find that values of *α* within the ferroelastic phase are more than an order of magnitude larger than within the paraelastic phase. In fact the NTE effect we observe, *α* = −407(28) MK^−1^ (20 < *T* < 240 K), is many times larger than the most extreme sustained NTE behaviour ever reported: *α* ≃ −170 MK^−1^ for FMOF-1,^16^*α* = −130 MK^−1^ for Ag_3_[Co(CN)_6_],^6^ and *α* = −80(12) MK^−1^ for the pillared zinc/terephthalate MOF of ref. 9. Consequently, *T*_f_ signifies not only the emergence of symmetry-breaking strain, but also a fundamental shift in the degree of framework flexibility.

In an attempt to explore the extent to which a similar transition might be brought about by guest uptake, we find that Cu(tcm) is reversibly convertible into a topologically-distinct solvate [Cu(MeCN)(tcm)] upon exposure to acetonitrile. Involving reconstruction between triply-interpenetrating (10,3)-*b* and interdigitated (6,3) nets, this solid–solid transformation highlights the extreme coordinative lability of Cu^+^ ions in the parent system. The two frameworks are distinguished not only by their network topology but also by their spectroscopic behaviour, suggesting that Cu(tcm) might find application as a solid-phase acetonitrile sensor.

## 2. Methods

All reagents were obtained from commercial suppliers and used as received.

### 2.1. Copper(i) tricyanomethanide

Synthesis of copper(i) tricyanomethanide followed the reduction protocol reported elsewhere for the related binuclear compound bis-(tricyanomethanido)-tetrakis-(triphenylphosphine)dicopper(i).^17^ A saturated aqueous solution of copper(ii) sulfate (Sigma Aldrich, 99%; 0.3991 g) was reduced by dropwise addition of a concentrated aqueous solution of excess sodium hydrogen sulfite (Sigma Aldrich, 0.3587 g). The resulting mint-green solution was stirred for 30 min, after which time an aqueous solution of a stoichiometric quantity of potassium tricyanomethanide (Alfa Aesar; 0.3229 g) was added dropwise. A fine, off-white precipitate formed immediately. After further stirring for 2 h, the precipitate was filtered, washed with H_2_O, and dried under vacuum to afford a polycrystalline sample of copper(i) tricyanomethanide.

Copper(i) tricyanomethanide could also be obtained by heating the acetonitrile complex (see below) under vacuum at 473 K for 12 h.

### 2.2. Acetonitrilocopper(i) tricyanomethanide

Polycrystalline samples of acetontrilocopper(i) tricyano-methanide were prepared by mixing acetonitrile solutions containing stoichiometric quantities of tetrakis(acetonitrilo)-copper(i) triflate (Sigma Aldrich, 0.9421 g) and potassium tricyanomethanide (Alfa Aesar, 0.3229 g). The reaction mixture was stirred for 2 h and the off-white precipitate filtered and then dried under vacuum to afford acetonitrilocopper(i) tricyanomethanide in quantitative yield.

Single crystal samples of acetonitrilocopper(i) tricyano-methanide could be obtained using slow-diffusion techniques. To separate arms of a glass H-tube were placed chilled acetonitrile solutions of potassium tricyanomethanide (Alfa Aesar, 47.18 mg) and tetrakis(acetonitrilo)copper(i) tetrafluoroborate (Sigma Aldrich, 320.0 mg). Further chilled acetonitrile was then carefully added until the cross-bar was filled. After a period of 14 d, small colourless single crystals suitable for single-crystal X-ray diffraction measurements had formed.

Acetonitrilocopper(i) tricyanomethanide could also be made by exposing dry copper(i) tricyanomethanide to a few drops of acetonitrile and leaving for 12 h, then filtering and drying at 50 °C under vacuum.

### 2.3. Single-crystal X-ray diffraction

Single-crystal X-ray diffraction data were collected for acetonitrilocopper(i) tricyanomethanide using an Agilent Technologies SuperNova diffractometer (Cu Kα radiation, *λ* = 1.5418 Å) at 100 K using an Oxford Cryosystems cryostream,^18^ scanning about the *ω* and *ϕ* circles. The data were integrated and corrected for absorption using CrysAlisPro, the structure was solved in *Pbca* using SIR92 (ref 19) and refined using CRYSTALS.^20^ All non-hydrogen atoms were refined anisotropically; hydrogen atom positions were refined against the low angle data, according to the procedure described in ref. 21, and were then constrained to ride on their host atoms. A final *R*-factor of 1.99% was obtained. Crystal structure refinement details are given in Table 1.

**Table 1:** Single-crystal X-ray diffraction data collection and refinement details for [Cu(MeCN)(tcm)] at *T* = 100 K

Formula	CuC_6_N_4_H_3_
Crystal size (mm)	0.033 × 0.034 × 0.039
Crystal symmetry	Orthorhombic
Space group	*Pbca*
*a* (Å)	12.9802(3)
*b* (Å)	8.14490(18)
*c* (Å)	14.5848(3)
*V* (Å^3^)	1541.94(5)
*Z*	8
No. of measured reflns.	14 516
No. of independent reflns.	1620
No. of observed rflns. [*I* > 2*σ*(*I*)]	1480
No. of parameters	100
*R*_int_	0.025
*R* (*I*/*σ* > 2.0)	0.0199
w*R*	0.0524
Δ*ρ*_max_, Δ*ρ*_min_ (*e* Å^−3^)	0.26, −0.29

### 2.4. Powder X-ray diffraction

All samples were assessed for crystallinity and purity *via* their X-ray powder diffraction patterns, measured using a Philips PW1730 diffractometer (Cu Kα radiation, *λ* = 1.541 Å) over the angular (2*θ*) range 10–70° using a step size of 0.05°. Higher-quality diffraction patterns were collected using a PANalytical X'Pert PRO diffractometer (monochromated Cu Kα_1_ radiation, *λ* = 1.54060 Å) using the same angular range and a step size of 0.008°.

Variable-temperature powder X-ray diffraction patterns were measured on a Siemens D5000 X-ray diffractometer (Cu Kα radiation, *λ* = 1.5418 Å) fitted with an Oxford Cryosystems PheniX cryostat. For temperatures below 300 K the copper(i) tricyanomethanide sample was combined with vaseline and then loaded into the sample holder. Data collections were carried out every 10 K on heating from 20 to 290 K. Scans were ~5 h long and were carried out over the angular range 5° < 2*θ* < 120° using a step size of 0.02°. Four minute intervals between data collections allowed for temperature equilibration of the sample.

All fits to X-ray powder diffraction data were carried out using TOPAS Academic;^22^ in general a modified Thompson–Cox–Hastings pseudo-Voigt (TCHZ) peak shape with a simple axial divergence correction was used and the large anisotropic strain found in this system was accounted for by using a Stephens anisotropic peak broadening term.^23^ Rietveld refinement of the room and low-temperature (20 K) structures of α- and β-Cu(tcm) were carried out. For the room temperature structure all atom positions were refined with appropriate bond distance (Cu–N, C–N and C–C) and bond angle restraints; the Cu isotropic atomic displacement parameter was refined freely and all atoms within the tcm moiety were constrained to have equal isotropic displacement parameters. A fourth-order spherical harmonic correction was used to account for preferred orientation. At low temperature, the tcm group was refined as a rigid body and all thermal parameters were fixed. Sample environment peaks were identified by their persistence across the α–β transition and were fitted using the Pawley method. Variable-temperature lattice parameters were determined by using sequential (seed-batch) Pawley fits to data in *Fdd*2 and *I*4_1_*md*, where the starting lattice parameters for each temperature were those found for the previous temperature.

### 2.5. Thermogravimetric analysis

Thermogravimetric measurements were carried out using a Perkin Elmer TGA 7, with Perkin Elmer TAC 7/DX thermal analysis controller, from 30 to 600 °C at a rate of 10 °C min^−1^ under flowing nitrogen.

### 2.6. Infrared spectroscopy

Infrared spectra were measured using a Bruker Tensor 27 FT-IR spectrometer fitted with an attenuated total reflectance diamond. A small amount of powdered sample was pressed onto the diamond surface and spectra were measured over the range 4000–600 cm^−1^. Background scans were recorded before each measurement. Absolute transmittance values were normalised to the tricyanomethanide C–N stretch (2200 cm^−1^) for each measurement.

## 3. Results and discussion

### Copper(i) tricyanomethanide structure

**Fig. 2:**
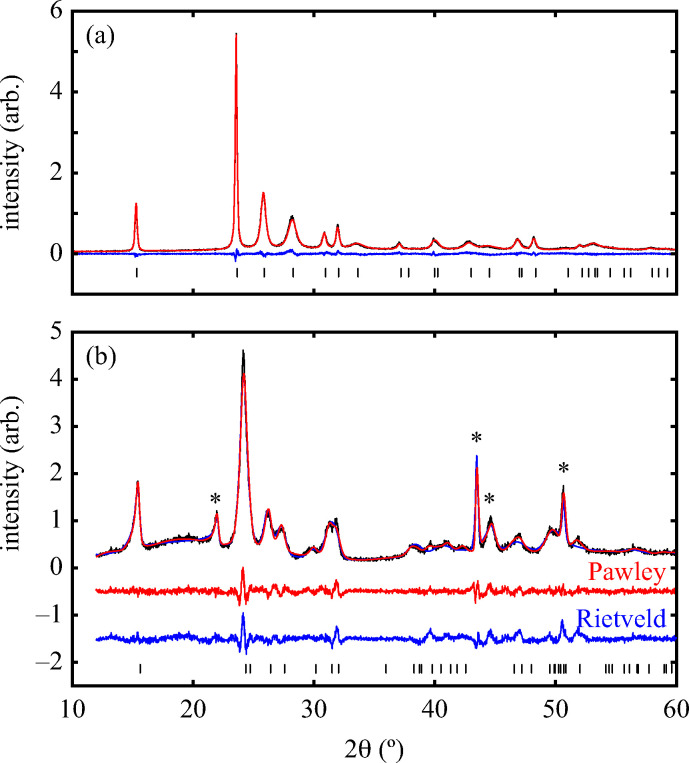
Powder X-ray diffraction data measured using Cu Kα radiation (black lines), with reflection positions indicated by vertical tick marks. (a) β-Cu(tcm), measured at 298 K; Rietveld fit (red lines), and difference (data – fit, blue lines). (b) α-Cu(tcm), measured at 20 K. Impurity peaks from the low-temperature sample environment are indicated by asterisks; both Pawley (red lines) and Rietveld (blue lines) fit and difference curves are shown, with the latter shifted vertically by 0.5 and 1.5 units. Pawley fits were used to extract thermal lattice parameter variations. The quality of the Rietveld fit is low as a result of severe anisotropic peak broadening and the large number of coupled positional degrees of freedom, but is sufficient to demonstrate the displacive nature of the phase transition.

**Fig. 3:**
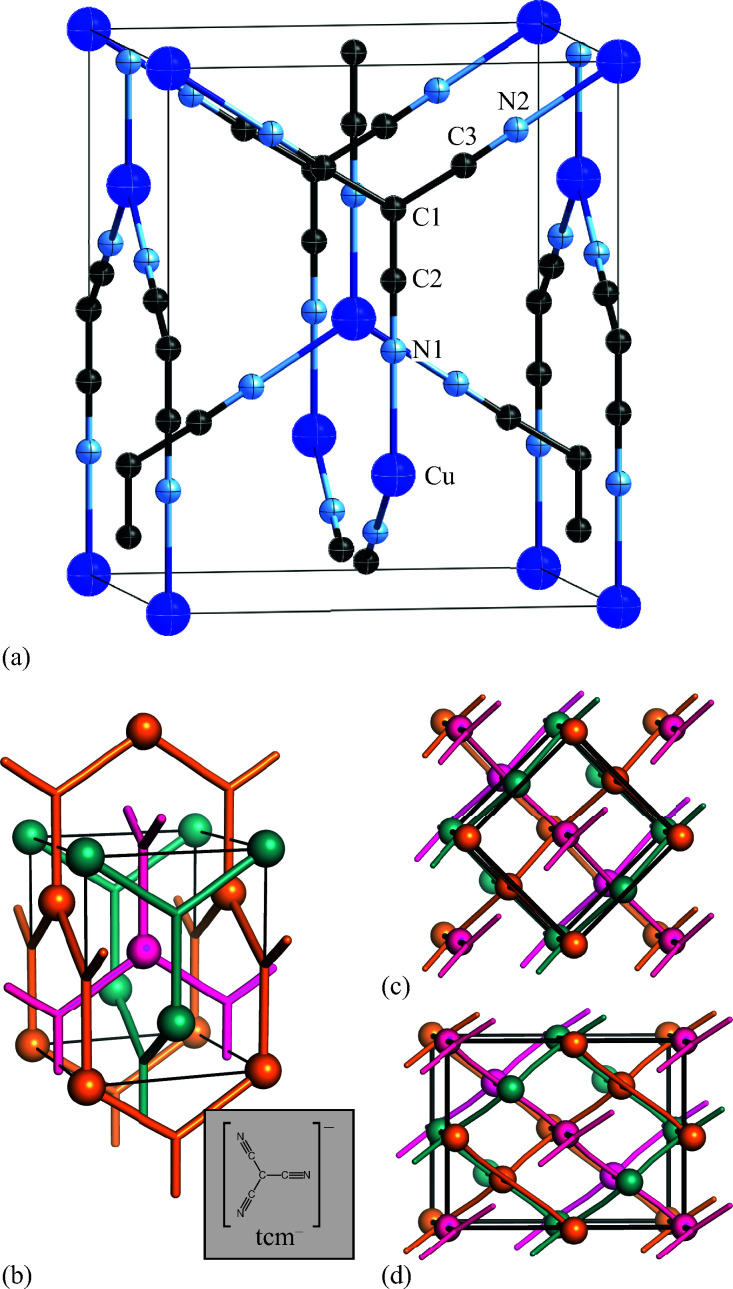
(a) Representation of the crystal structure of β-Cu(tcm), viewed with the tetragonal *c* axis oriented vertically. Thermal ellipsoids are represented at 50% probability. (b) Topological representation of the same framework, where Cu atoms are shown as large spheres, tcm moieties in stick representation, and the unit cell in black. The three mutually-interpenetrating (10,3)-*b* frameworks are coloured differently. The inset shows the chemical structure of the tricyanomethanide anion. (c) The same framework, viewed slightly away from *c*, illustrating its square wine-rack geometry with *θ* = 90°. (d) The low-temperature α-Cu(tcm) phase—shown here in terms of the structural model determined using powder X-ray diffraction data collected at 20 K—has the same network connectivity, but the loss of tetragonal symmetry allows *θ* to deviate from 90°. The *Fdd*2 unit cell, shown here in black, has twice the volume of the *I*4_1_*md* cell of β-Cu(tcm).

Having prepared a sample of Cu(tcm), its crystal structure was determined using powder X-ray diffraction methods. The ambient-temperature powder X-ray diffraction pattern—shown in Fig. 2(a)—could be indexed according to a tetragonal cell with lattice parameters *a* = 7.539(2) Å and *c* = 9.034(3) Å. A Pawley fit confirmed the space group assignment *I*4_1_*md*. Based on the cell metric and space group symmetry, a structural model was proposed in which both Cu atoms and tcm groups were centred on the 4*a* Wyckoff positions, with *z* = 0 for the former and *z* ≃ 
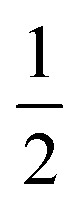
 for the latter. Using this initial model as a starting point, Rietveld refinement gave a satisfactory fit to data [Fig. 2(a)]. The corresponding atomic coordinates and atomic displacement parameters are given in Table 2, and the crystal structure is illustrated in Fig. 3(a–c). In this structure, both Cu^+^ and tcm^−^ ions are three-coordinate and connect to form a three-dimensional network with the (10,3)-*b* topology (Schläfli symbol 10^3^). This network is sufficiently open that three such nets interpenetrate to give a structure related to that of the MOF Δ-[Cr(Hbiim)(H_2_biim_2_)]SO_4_·H_2_O (biim = biimidazole).^24,25^

**Table 2:** Structural parameters for β-Cu(tcm) as determined by Rietveld refinement against powder X-ray diffraction data collected at 298 K. Space group: *I*4_1_*md*. Lattice parameters: *a* = 7.5510(4) Å, *c* = 9.0436(5) Å

Atom	*x*	*y*	*z*	*B*_iso_ (Å^2^)
Cu	0	0	0	4.30(7)
C1	0	0	0.4873(7)	1.44(9)
C2	0	0	0.3554(7)	1.44(9)
C3	0.1560(4)	0	0.56396(25)	1.44(9)
N1	0	0	0.2267(8)	1.44(9)
N2	0.2727(6)	0	0.6277(4)	1.44(9)

The established structural chemistry of copper(i) tricyanomethanides is limited to ternary and quaternary systems of the form [Cu(tcm)(N-donor)]·{guest}.^26^ This is perhaps surprising, given that the analogous silver(i) complex Ag(tcm) has been known for some time.^27^ The stronger preference for tetrahedral coordination over trigonal coordination for Cu^+^ relative to Ag^+^ may be an important factor at play in determining the different accessibilities of these two phases. It now seems that both Cu(tcm) and Ag(tcm) adopt polar crystal structures based on three-coordinate M(i)/tcm geometries, but in contrast to the three-dimensional topology of Cu(tcm) the structure of Ag(tcm) is described by stacked, pairwise-interwoven (6,3)-net layers.^27,28^ Despite our reliance here on powder diffraction methods for structure solution and refinement, the relatively small number of structural parameters and the good quality of fit-to-data give us confidence in the structural model we have determined for Cu(tcm). There is no risk of confusion with the Ag(tcm) structure, which would predict a very different powder diffraction pattern to that observed in our measurements. For completeness, we note that the Cu–N bond lengths and N–Cu–N bond angles we obtain (*d*(Cu–N) = 2.025, 2.036 Å; ∠(N–Cu–N) = 122.8, 114.5°) are fully consistent with the values reported elsewhere for [Cu(tcm)(hmt)], [Cu(tcm)(bipy)], and [Cu(tcm)(bpe)]·0.25bpe·0.5MeCN (hmt = hexamethylenetetramine; bipy = 4,4'-bipyridine; bpe = 1,2-bis(4-pyridyl)ethene): 1.92 < *d*(Cu–N) <2.22 Å and 103.3< ∠N–Cu–N <129.8°.^26^ Good consistency is of course expected given our use of soft Cu–N bond length restraints.

### Variable-temperature behaviour

**Fig. 4:**
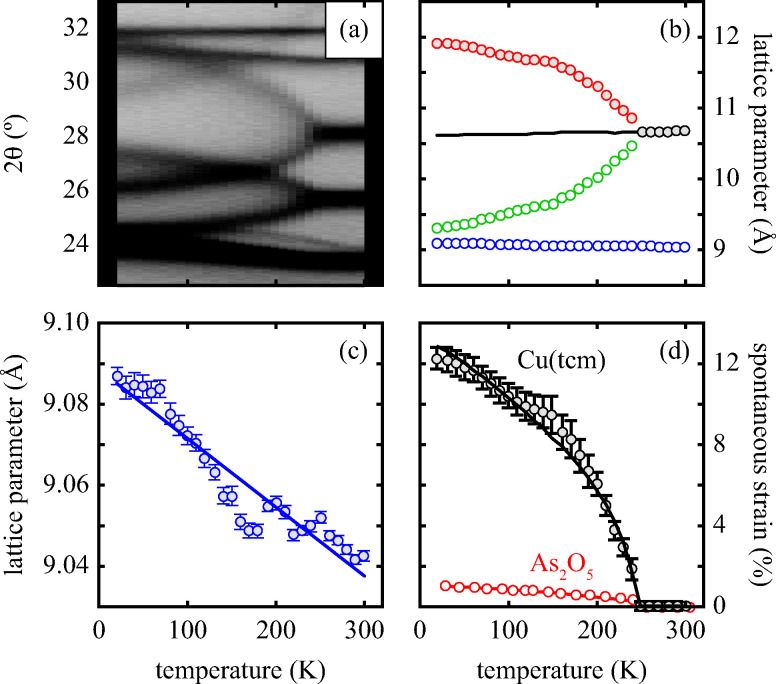
(a) Thermal variation in the X-ray powder diffraction pattern of Cu(tcm), shown here over a 2*θ* range that illustrates the large splitting of certain peaks at *T*_f_ = 240 K. (b) The corresponding thermal variation in lattice parameters. The parameters *a* and *b* are given by green and red circles, respectively, for the orthorhombic phase and black circles for the tetragonal phase; the *c* parameter is given by blue circles for both phases. Uncertainties are smaller than the symbols. The black line represents the mean value 
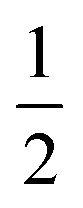
(*a* + *b*) for α-Cu(tcm). (c) The approximately-linear thermal variation of the *c* lattice parameter, as shown in (b) but given here on an expanded scale. (d) Thermal variation of the spontaneous strain as defined in eqn (2); the corresponding fit of form 
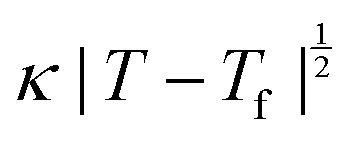
 is shown as a solid line. The strain observed for the “conventional” ferroelastic transition of As_2_O_5_ is shown in red, where the temperature scale has been shifted by 345 K in order to match transition temperatures.^30^

Powder X-ray diffraction patterns collected over the temperature range 20 < *T* < 290 K reveal a second-order phase transition near *T*_f_ = 240 K that involves splitting of a number of diffraction peaks in a manner consistent with a reduction from tetragonal to orthorhombic symmetry [Fig. 4(a)]. There are two maximal subgroups of *I*4_1_*md* with orthorhombic symmetry: namely, *Fdd*2 and *Imm*2. The reflection conditions differ between these two cases, and we found that our data were consistent only with the former. Making use of the appropriate cell transform1
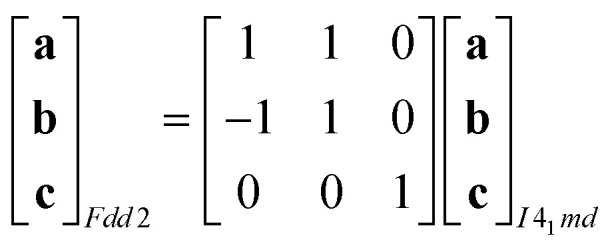
we obtained an approximate structural model for the low-temperature phase, from which Rietveld refinement was possible. The Rietveld fit obtained using data collected at 20 K is shown in Fig. 2(b). The quality of fit is satisfactory, though we note that severe anisotropic peak broadening associated with large lattice strains and also the experimental constraints of measuring diffraction data at 20 K have both degraded the fit relative to that achieved for data collected under ambient conditions. Because of the presence of anisotropic peak broadening, we have not explored the possibility of further symmetry lowering beyond *Fdd*2; nevertheless, any additional symmetry-lowering distortions—if present—must be very much weaker than the orthorhombic strain itself, which dominates the phase transition behaviour at *T*_f_. Moreover, because the low-angle peak intensities are well accounted for by the Rietveld model, we can be confident that the transition is displacive (*i.e.*, the framework connectivity is maintained). The transition from *I*4_1_*md* to *Fdd*2 is a ferroelastic transition associated with softening of the *Γ*_3_ zone-centre mode.^29^ The corresponding spontaneous strain2
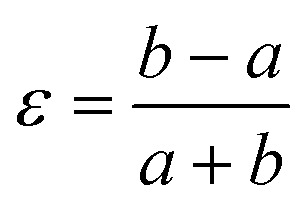
is expected on the basis of Landau theory to vary as 
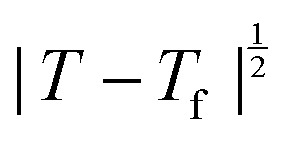
 near *T*_f_ (*i.e.*, the transition should be second order). Experimental values of *ε* determined from our lattice parameter data are shown in Fig. 4(d) and are consistent with this expected temperature dependence, indicating that this is a proper (*i.e.* strain-driven) ferroelastic transition.^13,30^ A combination of spectroscopic measurements and lattice dynamical modelling would help determine the microscopic driving force for *Γ*_3_ mode softening. Here we note only that this mode allows tilting of the tcm units about an axis parallel to *c*, which has the effect of reducing slightly the distance between tcm groups of one framework and Cu^+^ centres of the other two interpenetrated frameworks. Consequently, at low temperatures there may be a small electrostatic driving force for symmetry lowering that is overcome by the increased vibrational entropy of the *I*4_1_*md* geometry only for temperatures above *T*_f_.

The atomic coordinates and atomic displacement parameters determined from our Rietveld refinement for the low-temperature phase are given in Table 3, and the crystal structure itself is illustrated in Fig. 3(d). From this structural model it is clear that the framework connectivity is entirely preserved across *T*_f_. The single most important difference between high- and low-temperature phases of Cu(tcm) is the framework angle *θ*: in the former it is identically equal to 90° whereas in the latter it is reduced to *θ* = 2 tan^−1^
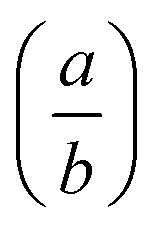
 = 76° at 20 K. Noting the parallel to symmetry-breaking transitions in various well-known framework silicates such as cristobalite and quartz, we borrow from the terminology of that field and denote the high-temperature phase as β-Cu(tcm) and the low-temperature phase as α-Cu(tcm).

**Table 3:** Structural parameters for α-Cu(tcm) as determined by Rietveld refinement against powder X-ray diffraction data collected at 20 K. Space group: *Fdd*2. Lattice parameters: *a* = 9.297(5) Å, *b* = 11.891(5) Å, *c* = 9.093(5) Å. All displacement parameters were fixed at *B*_iso_ = 2 Å^2^

Atom	*x*	*y*	*z*
Cu	0	0	0
C1	0	0	0.503(3)
C2	0	0	0.350(3)
C3	−0.075(4)	0.082(5)	0.579(3)
N1	0	0	0.229(3)
N2	−0.136(5)	0.149(6)	0.641(3)

Access to variable-temperature diffraction data allows us to explore the mechanical flexibility of both α- and β-Cu(tcm) in terms of their linear coefficients of thermal expansion, and so to determine whether the intuitive picture developed in Fig. 1 does indeed hold in practice. For β-Cu(tcm) there are two independent coefficients of thermal expansion, corresponding to the behaviour parallel and perpendicular to the tetragonal *c* axis:3
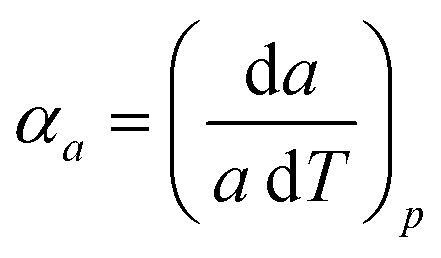
4
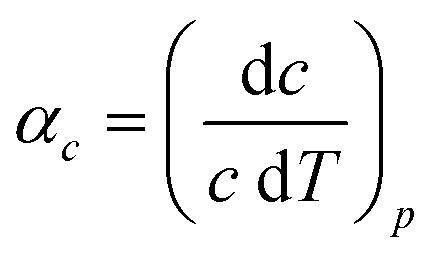


Our data give *α*_*a*_ = +10.8(23) MK^−1^ and *α*_*c*_ =−21(4) MK^−1^,^31^ which—although anomalous in sign and magnitude for conventional framework materials (*a* ≃ 10 MK^−1^)—are comparable to the values obtained for many MOFs.^32–36^ In such systems the low energies of transverse vibrational modes tend to favour NTE, which then often drives anomalously large PTE in other crystal directions.^32^ Within the low-temperature α-Cu(tcm) phase, the behaviour along *c* is essentially unchanged: here we find *α*_*c*_ = −13.6(11) MK^−1^ over the temperature range 20–240 K [Fig. 4(c)]. In contrast, the behaviour along the *a* and *b* axes is fundamentally different from that observed in β-Cu(tcm) because these are the axes affected by changes in the framework angle *θ*. Linear fits to our data over the same temperature range 20–240 K give *α*_*a*_ = +591(28) MK^−1^ and *α*_*b*_ = −407(28) MK^−1^. There is clearly a drastic increase in thermal expansivity—and hence framework flexibility—on cooling below *T*_f_, justifying our characterisation of the α/β-phase transition as a ‘flexibility transition’ in Cu(tcm).

The extraordinarily large coefficients of thermal expansion observed here for α-Cu(tcm) deserve further comment because they exceed even the threshold for so-called ‘colossal’ NTE and PTE (|*α*| = 100 MK^−1^).^6^ Examples of colossal thermal expansion remain extremely rare, even amongst MOFs. Until recently, ‘FMOF-1’ exhibited the strongest known thermal expansion behaviour: its coefficients of thermal expansion are *α*_*a*_ = +230 MK^−1^ and *α*_*c*_ = −170 MK^−1^.^16^ More extreme PTE has since been observed in the zinc terephthalate-based MOFs of ref. 9 for which *α*_*b*_ = +340(35) MK^−1^ over the temperature range 303–463 K.^31,37^ Yet the response we see here for α-Cu(tcm) is larger again by some margin; in particular, the uniaxial NTE along *b* is so extreme that an aligned composite containing just 2.5% Cu(tcm) would be sufficient to counteract the PTE effect of conventional engineering materials. Moreover, the temperature range over which this NTE occurs (20–240 K)—perhaps problematic for some applications—is nonetheless relevant to industries such as aerospace.

But why is α-Cu(tcm) so very flexible? Empirically it is found that framework flexibility tends to scale inversely with metal–ligand interaction strength.^32,38,39^ Here, the low charge densities of Cu^+^ and of the terminal N atoms of the tcm^−^ ion^40^ will reduce any electrostatic contribution to the thermodynamic cost of framework flexing. Furthermore the combination of a closed-shell d^10^ configuration together with low Cu 3d–tcm π^*^ overlap will mean that (directional) covalent effects are also much smaller than in many other MOFs. Consequently, the mechanical properties of this system would be expected to be especially sensitive to low-energy interactions such as dispersion forces, which are well-known to drive extreme thermal expansion in other framework materials.^6^ Quantum mechanical calculations would help understand more fully the origin of this extreme mechanical flexibility.^4,5,41^

### Solvent-induced reorganisation

**Fig. 5:**
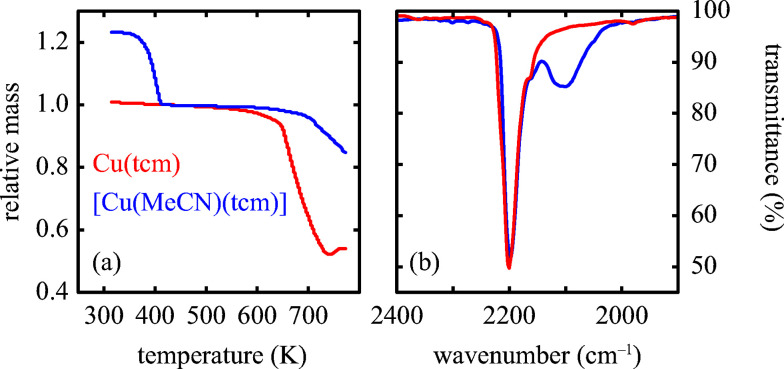
(a) Thermogravimetric behaviour of Cu(tcm) and [Cu(MeCN)(tcm)]. The mass loss observed near 400 K for the latter corresponds to volatilisation of one molar equivalent of MeCN. Both TGA curves have been normalised to the mass measured at 420 K (b). High-frequency region of the infrared absorption spectra of Cu(tcm) and [Cu(MeCN)(tcm)] showing the emergence of an additional band in the latter, attributed to the nitrile C–N stretch. Colour scheme as in (a).

**Fig. 6:**
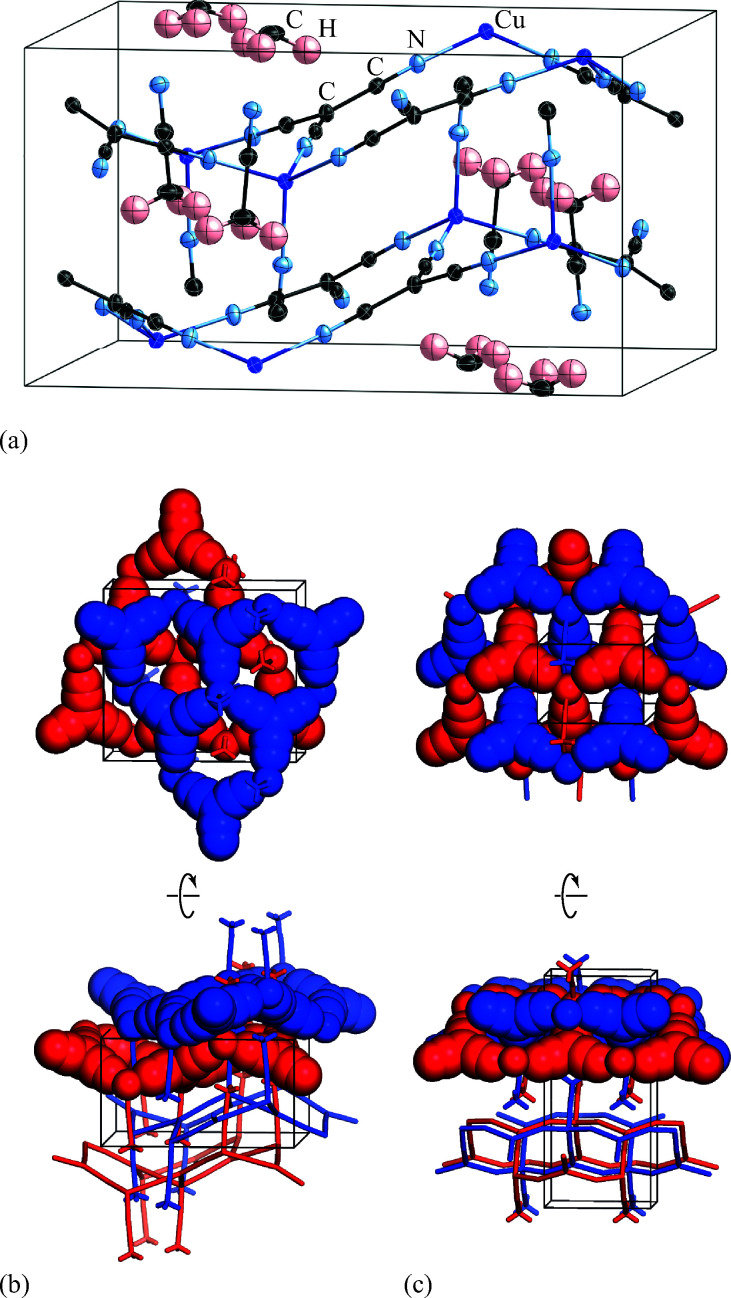
(a) Representation of the crystal structure of [Cu(MeCN)(tcm)], viewed away from **a**, as determined using single-crystal X-ray diffraction measurements carried out at 100 K. Thermal ellipsoids are drawn at 50% probability level. Panels (b) and (c) show topological representations of, respectively, this same framework and that of [Ag(MeCN)(tcm)] as reported in ref. 42. Both structures are assembled from honeycomb (6,3) nets of connected Cu/Ag cations and tcm^-^ anions. In (b) these nets stack such that the MeCN ligands of one net interdigitate the hexagonal channels of the next. In (c) pairs of adjacent nets interweave, with the MeCN groups occupying the space between adjacent honeycomb pairs. The corresponding unit cells for both systems are shown in black; the Cu/Ag and tcm components of two adjacent honeycomb nets (but not the coordinated MeCN groups) are shown in space-filling representation.

Exposure of a polycrystalline sample of Cu(tcm) to acetonitrile gave rise to a new phase with a powder diffraction profile quite distinct from that of either α- or β-Cu(tcm). TGA measurements of this phase revealed a mass loss of 19% at 400 K, which suggests the formulation [Cu(MeCN)(tcm)] (expected mass loss 21%) [Fig. 5(a)]. We found that single-crystal samples could in fact be obtained directly from acetonitrile solution, which allowed us to determine the crystal structure using single-crystal X-ray diffraction methods. Our structure solution is illustrated in Fig. 6(a), confirming the formulation [Cu(MeCN)(tcm)]; key structural parameters are given as ESI.† In this structure, the Cu^+^ ions now have tetrahedral coordination, with one coordination site occupied by a MeCN group; the tcm^−^ ion remains trigonal in its coordination. From a topological perspective, both Cu and tcm components are effectively three-coordinate, connecting to form (6,3) honeycomb layers (Schläfli symbol 6^3^). These layers stack without interpenetration, in contrast to the related acetonitrile derivative [Ag(MeCN)(tcm)] reported elsewhere [Fig. 6(b–c)].^42^

**Fig. 7:**
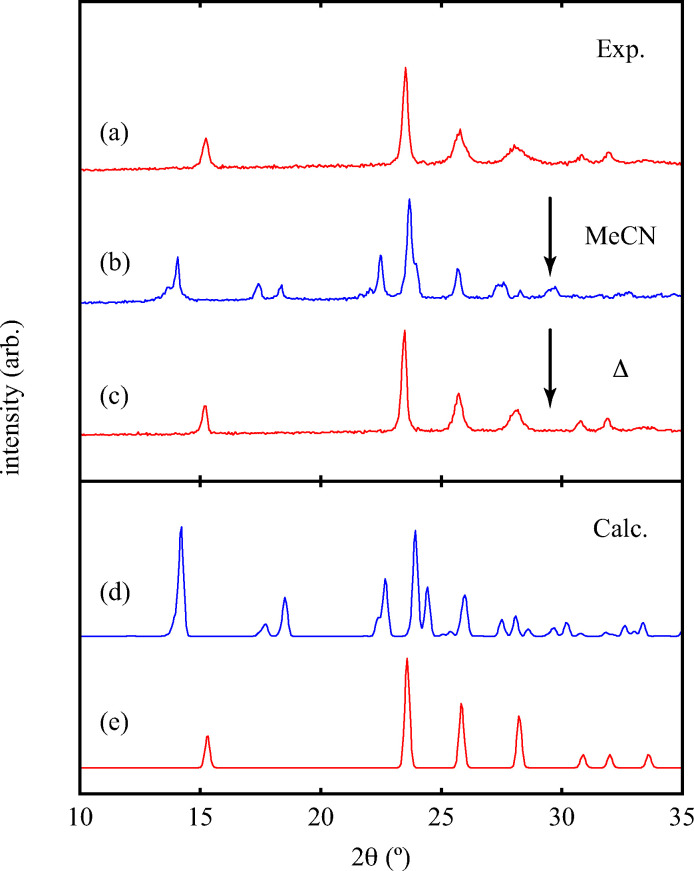
(Top) Powder X-ray diffraction patterns of (a) as-prepared Cu(tcm), (b) the same sample after exposure to MeCN, and (c) after subsequent heating at 200 °C for 3 h. (Bottom) Calculated X-ray diffraction patterns of (d) [Cu(MeCN)(tcm)] and (e) Cu(tcm).

Of greatest relevance to this study is the clear difference in connectivity between the structures of Cu(tcm) and [Cu(MeCN)(tcm)]. Generation of the latter by exposure of the former to acetonitrile must proceed by concerted cleavage and reconstruction of Cu–tcm bonds, which in itself demonstrates a remarkable coordinative flexibility of the original framework. The closed-shell d^10^ electronic configuration of Cu^+^ facilitates bond cleavage, and allows the formation of network structures with coordination numbers between two and four.^43,44^ Moreover, this interconversion process is reversible: powder samples of [Cu(MeCN)(tcm)]—themselves prepared by exposing Cu(tcm) to MeCN—revert to β-Cu(tcm) on heating at 200 °C for 3 h [Fig. 7]. This series of solid–solid transformations can be followed using infrared spectroscopy, with the acetonitrile-containing phase exhibiting an additional characteristic absorption at 2101 cm^−1^ associated with the nitrile C–N stretch [Fig. 5(b)]. Samples of Cu(tcm) exposed to moist air over extended periods show no structural transformation, so the binding of MeCN appears to be strongly selective even in the presence of competitive ligands such as H_2_O. This observation suggests that, like the recently-reported CuCN-based systems of ref. 45, and quite separate from its extreme thermal expansion behaviour, Cu(tcm) may find application in acetonitrile sensing.

## 4. Conclusions

In summary, the ‘wine-rack’-type framework material Cu(tcm) exhibits a ferroelastic transition at 240 K. This transition demarcates an order-of-magnitude change in elastic response, seen here most obviously in the ‘colossal’ PTE and NTE effects of α-Cu(tcm) that disappear in β-Cu(tcm). Indeed this anomalous thermal expansion behaviour establishes a new record within the field that means that Cu(tcm) may find application in the athermalisation of functional composites.

With respect to the ferroelastic transition itself, there are strong qualitative parallels to well-known tetragonal/orthorhombic transitions in oxide frameworks such as As_2_O_5_,^30^ for which the elastic anomalies associated with ferroelasticity affect not only the thermal expansion, but also the lattice dynamics (*e.g.* mode softening, phonon lifetimes), microdomain formation, twin-wall motion, and susceptibility. While our study has been limited to determination of the thermal variation in lattice parameters, we anticipate that further investigation of elastic behaviour in Cu(tcm) at *T*_f_ may prove informative in establishing parallels between conventional and MOF-type framework materials.^14,46^ In particular, the verification of softening of zone-centre acoustic phonons polarised along 〈110〉, and the use of microscopy techniques to characterise domain microstructure within the low-temperature ferroelastic phase will provide important experimental constraints on the phenomenology of what appears to be a canonical proper ferroelastic transition in a MOF. The most obvious difference between the ferroelastic behaviour of Cu(tcm) and that of “conventional” systems such as As_2_O_5_ is the very large strains realised within the ferroelastic phase [Fig. 4(d)], as implied by the colossal thermal expansion.

Finally, the coordinative flexibility of Cu(tcm) is found to affect not only elastic behaviour but also the ability of the framework to reorganise itself on exposure to a coordinating solvent. In this way, we have demonstrated proof-of-principle for application of Cu(tcm) as an acetonitrile sensor. Given the diversity of known Ag(tcm)·{guest} phases,^27,42^ the possibility that Cu(tcm) may respond selectively to a variety of different solvents offers an attractive avenue for further research.

## Supplementary Material

CE-017-C4CE01572J-s001

CE-017-C4CE01572J-s002
