# Supplementary material for: Flexibility transition and guest-driven reconstruction in a ferroelastic metal–organic framework
Source: CrystEngComm. 2014 Oct 2;17(2):361–9. doi: 10.1039/c4ce01572j (PMC4304274; doi:10.1039/c4ce01572j)
Supplement: CE-017-C4CE01572J-s001 [file CE-017-C4CE01572J-s001.pdf]

Electronic Supplementary Material (ESI) for CrystEngComm.
This journal is © The Royal Society of Chemistry 2014

Flexibility Transition and Guest-Driven Reconstruction in a Ferroelastic Metal–Organic Framework

SUPPLEMENTARY INFORMATION

Sarah J. Hunt, Matthew J. Cliffe, Joshua A. Hill, Andrew B. Cairns, Nicholas P. Funnell, and Andrew L. Goodwin*

Department of Chemistry, University of Oxford, Inorganic Chemistry Laboratory, South Parks Road, Oxford OX1 3QR, U.K.

*To whom correspondence should be addressed; e-mail: andrew.goodwin@chem.ox.ac.uk.

Submitted to CrystEngComm

1 Single-crystal X-ray structural parameters

Table S1: Structural parameters for [Cu(MeCN)(tcm)] as determined by refinement against single-crystal X-ray diffraction data collected at 100 K. Space group: *Pbca*. Lattice parameters: a = 12.9802(3) Å, b = 8.14490(18) Å, c = 14.5848(3) Å. Estimated standard errors are given in parentheses.

Atom	x	y	z	U_{eq} (Å^2)
Cu	0.588127(13)	0.44049(2)	0.628675(12)	0.0136
C11	0.69527(9)	0.32962(15)	0.81154(8)	0.0159
C12	0.86036(9)	0.32301(15)	0.88643(8)	0.0148
C13	0.70950(9)	0.20812(15)	0.96428(8)	0.0141
C14	0.75490(9)	0.28398(16)	0.88784(8)	0.0151
C21	0.59224(10)	0.82529(18)	0.64049(9)	0.0210
C22	0.59681(13)	1.00325(18)	0.65007(12)	0.0312
N11	0.64984(8)	0.36967(15)	0.74738(7)	0.0193
N12	0.94616(8)	0.35831(15)	0.88161(7)	0.0176
N13	0.67102(8)	0.14601(14)	1.02642(7)	0.0169
N21	0.58913(9)	0.68611(16)	0.63311(8)	0.0212
H221	0.5567	1.0371	0.7002	0.0491
H222	0.6655	1.0361	0.6569	0.0502
H223	0.5733	1.0534	0.5950	0.0478

2 Variable-temperature lattice parameters

Table S2: Variable-temperature lattice parameters for Cu(tcm) as determined using powder X-ray diffraction. The estimated thermal stability is σT = 0.1 K in each case and all other estimated standard errors are given in parentheses.

T (K)	a (Å)	b (Å)	$\frac{1}{2}(a+b)$ (Å)	c (Å)
20	9.314(3)	11.916(4)	10.615(3)	9.087(2)
30	9.321(3)	11.907(5)	10.614(3)	9.084(3)
40	9.340(3)	11.887(5)	10.614(3)	9.085(3)
50	9.364(3)	11.874(5)	10.619(3)	9.084(3)
60	9.381(3)	11.863(5)	10.622(3)	9.083(3)
70	9.424(3)	11.828(5)	10.626(3)	9.084(2)
80	9.455(3)	11.789(5)	10.622(3)	9.078(3)
90	9.487(3)	11.756(5)	10.622(3)	9.075(2)
100	9.518(3)	11.729(5)	10.624(3)	9.072(2)
110	9.555(3)	11.706(5)	10.630(3)	9.070(2)
120	9.582(3)	11.685(6)	10.633(3)	9.066(2)
130	9.604(4)	11.674(6)	10.639(3)	9.063(2)
140	9.626(4)	11.668(6)	10.647(4)	9.057(2)
150	9.640(4)	11.650(7)	10.645(4)	9.057(2)
160	9.739(4)	11.567(7)	10.653(4)	9.0509(19)
170	9.776(5)	11.539(8)	10.658(4)	9.0488(18)
180	9.860(4)	11.447(6)	10.653(4)	9.0487(18)
190	9.944(4)	11.364(6)	10.654(4)	9.0548(14)
200	10.014(3)	11.301(5)	10.658(3)	9.0556(17)
210	10.124(3)	11.188(4)	10.656(2)	9.0534(16)
220	10.251(3)	11.050(4)	10.651(2)	9.0477(14)
230	10.348(3)	10.970(3)	10.659(2)	9.0489(13)
240	10.466(4)	10.859(4)	10.663(3)	9.0499(15)
250	10.6658(16)	-	-	9.0520(13)

Continued on next page

Table S2 – continued from previous page

T (K)	a (Å)	b (Å)	$\frac{1}{2}(a + b)$ (Å)	c (Å)
260	10.6700(16)	-	-	9.0476(13)
270	10.6698(15)	-	-	9.0464(12)
280	10.6693(15)	-	-	9.0441(12)
290	10.6721(15)	-	-	9.0416(12)
300	10.6727(15)	-	-	9.0425(12)
